# First-Principles Study of High-Pressure Phase Stability and Electron Properties of Be-P Compounds

**DOI:** 10.3390/ma15031255

**Published:** 2022-02-08

**Authors:** Han Liu, Yaqian Dan, Ao Zhang, Siyuan Liu, Jincheng Yue, Junda Li, Xuejiao Ma, Yanping Huang, Yanhui Liu, Tian Cui

**Affiliations:** 1 Institute of High Pressure Physics, School of Physical Science and Technology, Ningbo University, Ningbo 315211, China; hanliu96@sina.com (H.L.); danyaqian@nbu.edu.cn (Y.D.); Ao_zhang1997@163.com (A.Z.); augus_jincheng@sina.com (J.Y.); Linkim665@163.com (J.L.); huangyanping@nbu.edu.cn (Y.H.); 2 Department of Physics, College of Science, Yanbian University, Yanji 133000, China; 3 School of Physics, Southeast University, Nanjing 211189, China; syliu@seu.edu.cn; 4 Science and Technology on Transient Impact Laboratory, No. 208 Research Institute of Ordnance Industries, Beijing 102202, China; maxj_2020@163.com

**Keywords:** phase transition, structural prediction, high pressure, first-principles

## Abstract

New, stable stoichiometries in Be-P systems are investigated up to 100 GPa by the CALYPSO structure prediction method. Along with the BeP_2_-*I*4_1_/*amd* structure, we identify two novel compounds of Be_3_P_2_-*P*-42_1_*m* and Be_3_P_2_-*C*2/*m*. It should be noted that the Be-P compounds are predicted to be energetically unfavorable above 40 GPa. As can be seen, interesting structures may be experimentally synthesizable at modest pressure. Our results indicate that at 33.2 GPa, the most stable ambient-pressure tetragonal Be_3_P_2_-*P*-42_1_*m* transitions to the monoclinic Be_3_P_2_-*C*2/*m* structure. Moreover, the predicted Be_3_P_2_-*P*-42_1_*m* and Be_3_P_2_-*C*2/*m* phases are energetically favored compared with the Be_3_P_2_-*Ia*-3 structure synthesized experimentally. Electronic structure calculations reveal that BeP_2_-*I*4_1_/*amd*, Be_3_P_2_-*P*-42_1_*m*, and Be_3_P_2_-*C*2/*m* are all semiconductors with a narrow band gap. The present findings offer insight and guidance for exploration toward further fundamental understanding and potential applications in the semiconductor field.

## 1. Introduction

An important part of computational materials science is predicting novel forms of materials and describing their different characteristics, which are influenced by their electronic structures. Narrow-gap semiconducting substances are an important material with a number of applications, including lasers, infrared detectors, ultrasonic multipliers, and solar cells [1], electrically driven light sources [2], magnetic sensors [3], and thermophotovoltaic cells [4]. Although the III–V group phosphides and nitrides [5] have received the most attention, the II–V group compounds are also being investigated for optoelectronic applications [6,7,8]. Surprisingly, these compounds have abundant semiconductor properties [9,10,11] and crystallize in several phases [12,13]. The electronic structure of Mg_3_P_2_, as a II–V group compound, has been investigated by first principles. This compound has a straight bandgap of 1.73 eV, according to researchers [14]. There is ongoing debate over the stoichiometric composition of Ca_3_P_2_, and an experimental study on the thermodynamic properties of this compound has been published [15].

However, compared with their calcium and magnesium counterparts, beryllium phosphides have received little theoretical or experimental attention [16,17,18,19,20,21]. It is significant to recognize the crystal structures of any material in order to understand their physical and chemical properties, as well as their practical uses. Up to now, previous studies have demonstrated lattice constants of the anti-bixbyite structure of Be_3_P_2_ [20,21]. Regarding the tetragonal structure of Be_3_P_2_, based on structural refinements of X-ray and neutron diffraction data, Elmaslout reported lattice constants and structure factors [22]. According to Carvalho et al., Be_3_P_2_ microcrystals arise in Be-doped phosphorus-based semiconductor compounds generated by CBE (chemical beam epitaxy) in Be-rich environments and at temperatures over 500 °C [23]. Nevertheless, for semiconductor applications, high pressure phases are paramount. More than 90 percent of the matter in nature is under high pressure. As the pressure increases, the distance between atoms or molecules in condensed matter will gradually decrease, leading to an increase in the number of electron orbitals overlapping between adjacent atoms, which often leads to changes in the physical properties of the material itself. As a result, it is of the utmost importance to perform a thorough investigation of the crystal structure with varied beryllium phosphide stoichiometries and to explore the associated bonding properties under pressure.

In the present paper, utilizing first-principles swarm-intelligence structure search, we explored the binary Be-P phase diagram and built a complete understanding of its crystal structure evolution in the range from 0 to 100 GPa. Under environmental conditions, successfully reproduced BeP_2_-*I*4_1_/*amd* and Be_3_P_2_-*Ia*-3 structures have been reported. In particular, for Be_3_P_2_ at ambient pressure, we predict a more favorable Be_3_P_2_-*P*-42_1_*m* tetragonal structure than the experimentally synthesized structure of Be_3_P_2_-*Ia*-3. On the contrary, it can be discovered from the analysis of the calculation results that Be_2_P, BeP, and BeP_3_ are predicted to dissociate into Be and P under the pressure in our scheme. In the subsequent work, we provide a detailed discussion of the methods of our calculations and the results obtained, including structural parameters, electronic band structure, density of states (DOS), and bonding character of the beryllium phosphide systems. Our results are of great significance for the further study of the structures and properties of Be-P system under high pressures.

## 2. Computational Details

The structure search for the potential BeP*_x_* (x = 1/2, 2/3, and 1–3) compounds under the pressure range of 0–100 GPa was carried out utilizing the CALYPSO code’’s particle swarm optimization methodology [24,25,26]. Several recent effective uses of this technology include structure predictions on various crystalline systems, as well as determining stable or metastable structures based on chemical composition [27,28,29,30]. The exchange-correlation potentials were handled using the generalized gradient approximation (GGA) of Perdew–Burke–Ernzerh (PBE) [31,32]. The underlying optimizations were carried out utilizing the Vienna ab initio simulation (VASP) 5.4.1 software [33,34] and projector-augmented plane wave (PAW) [35] potentials with a 600 eV energy cutoff. The valence states of the Be and P potentials are 2s^2^ and *3*s^2^3p^3^, respectively. We employed the Monkhorst-Pack approach for the Brillouin region integral and tested the convergence of the ground state computations using consistently increasing *k*-point gridding for the considered structures. In order to obtain total energy astringency below 1 meV/atom, Monkhorst-Pack k-point grid is selected as 2π × 0.025 Å^−1^ [36,37]. Phonon dispersion curves are generated using a finite displacement approach in the PHONOPY program [38] to verify that the structures in the Be-P system are dynamically stable. The projector augmented wave (PAW) pseudopotential employing PBE and the band structures of Be-P phases are computed using the Heyd–Scuseria–Ernzerhof (HSE) hybrid functional, in order to evade the innate weakness of GGA when handling the band structures of semiconducting materials [39].

## 3. Results

### 3.1. Crystal Structure

**Figure 1:**
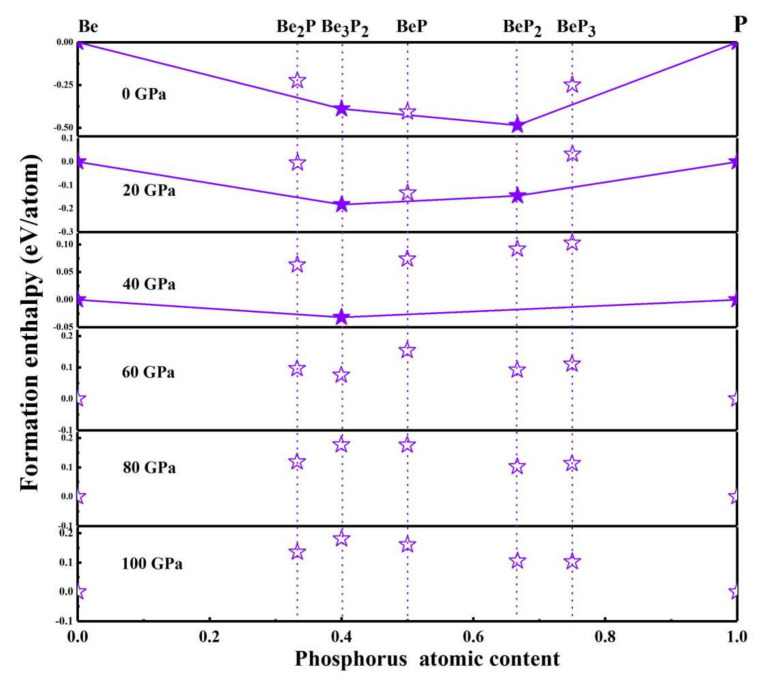
(color online). In relation to elemental beryllium and phosphorus solids, relative enthalpies of creation of the Be−P phase. Solid lines connect the stable phases to form convex shells (solid stars). Open stars indicate the unstable/meta stable phases.

Through the emulation of the variable unit with cell sizes of 1–4 formula units (f.u.), we predicted the structures of BeP*_x_* (x = 1/2, 2/3, and 1–3) in the range from 0 to 100 GPa. Then, the structures’ relative energetic stabilities are determined by calculating the formation enthalpy (Δ*H_f_*) with respect to elementary Be and P solids using the following formula: Δ*H_f_* = [*H*(BeP*_x_*) − *H*(Be) − x*H*(P)]/(x + 1). Here, *H*(BeP*_x_*) represents the enthalpy of the predicted compound and *H*(Be) and *H*(P) express the enthalpy of elemental Be and P, respectively. Δ*H_f_* is the computed formation enthalpy for the energetically advantageous beryllium phosphides. Figure 1 describes the enthalpies of formation of the predicted structure under varied pressures. For reference, the elemental Be solid with *P*6_3_/*mmc* symmetry, within their respective stable pressure range and the hexagonal, simple cubic, and triclinic structures for P, were used. The Δ*H_f_* for the Be-P compounds was calculated only for the lowest energy obtained structures of each stoichiometry. A Be-P compound was designated to be “stable” (in comparison with the solid elements) only when the lowest formation enthalpy is negative, while a designated “metastable” system was one discovered above the convex hulls. That is to say, it Δ*H_f_* value was smaller than the sum of the two elemental products′ Δ*H_f_* values. A given compound is stable if it has a positive enthalpy of decomposition when converted into other compounds, as described by the convex hulls. In ambient conditions, it is not difficult to discover that only Be_3_P_2_ and BeP_2_ compounds are stable. In contrast, the calculations show that BeP*_x_* (x = 1/2, 2/3, and 1–3) are unstable over 40 GPa. Subsequently, we shine a spotlight on the stable compounds. 

**Figure 2:**
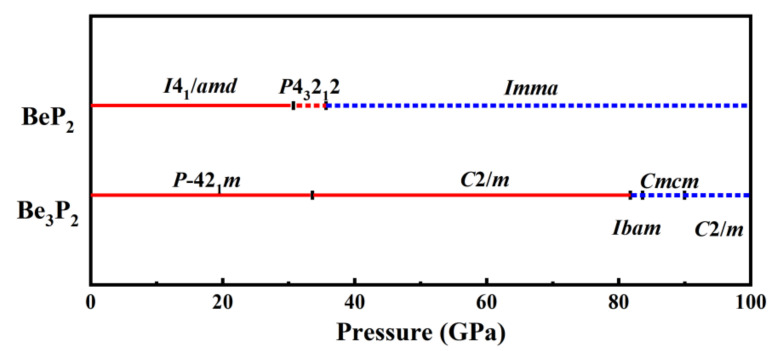
(color online). Be−P compounds composition-pressure phase graph. The metallic and insulating phases are represented by blue and red, respectively. Stable (metastable) phases are represented by solid (dashed) lines.

Figure 2 depicts a complete composition–pressure phase graph of the Be-P system and identifies stable structures by colors and space groups. The Be_3_P_2_-*P*-42_1_*m* and BeP_2_-*I*4_1_/*amd* are stable compositions at ambient pressure. Then, one more new stoichiometric Be_3_P_2_-*C*2/*m* becomes stable at 33.2 GPa. This finding for BeP_2_-*I*4_1_/*amd* is consistent with a previous study [40]. Interestingly, for Be_3_P_2_ at ambient pressure, the predicted Be_3_P_2_-*P*-42_1_*m* tetragonal structure is the most stable structure that has not been experimentally synthesized at ambient pressure. Moreover, the enthalpy of Be_3_P_2_-*Ia*-3 synthesized by experiment [18,19] has higher energy than our predicted structures of Be_3_P_2_-*P*-42_1_*m*, and even higher energy than the Be_3_P_2_-*C*2/*m* phase under high pressure. To explore the phase behavior of Be_3_P_2_ and BeP_2_ under high pressure, we investigate all the structures in the pressure range.

**Figure 3:**
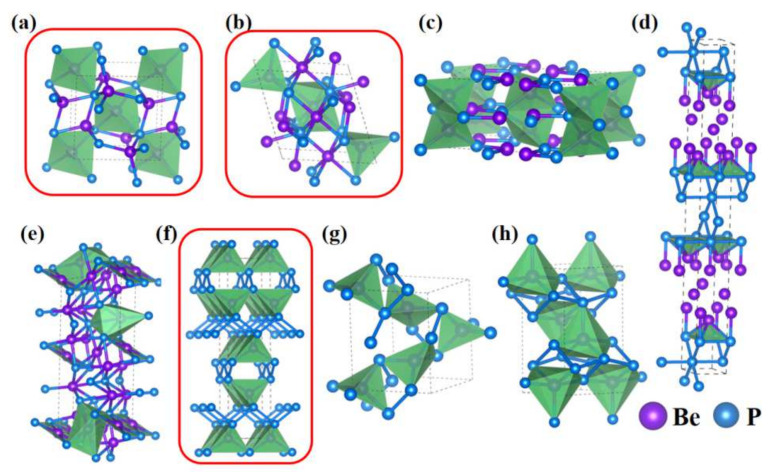
(color online). Crystal structures of the predicted Be−P system for (**a**) Be_3_P_2_−*P*−42_1_*m*, (**b**) Be_3_P_2_−*C*2*/m*, (**c**) Be_3_P_2_−*Ibam*, (**d**) Be_3_P_2_−*Cmcm*, (**e**) Be_3_P_2_−*C*2*/m*, (**f**) BeP_2_−*I*4_1_*/amd*, (**g**) BeP_2_−*P*4_3_2_1_2, and (**h**) BeP_2_−*Imma*. Be atoms and P atoms are represented by the purple and blue spheres, respectively. Inside the red circular region, the structures are stable.

Here, we present a systematic analysis of atomic arrangements and structural characteristics for Be_3_P_2_ and BeP_2_. In order to describe a phase transition under high pressure, we list all structures for 0–100 GPa, including the metastable structures. For Be_3_P_2_, at ambient pressure, the lattice parameters of Be_3_P_2_-*P*-42_1_*m* are *a* = *b* = 5.802 Å, *c* = 3.830 Å (Figure 3a), which has tetragonal primitive symmetry. In the unit cell, six Be atoms occupy the Wyckoff 4*e* (0.639, 0.139, 0.247) and 2*b* (0.500, 0.500, 0.500) sites, and four P atoms are located in the 4*e* (0.700, 0.270, 0.760) sites. Each Be atom is connected with four P atoms forming a tetrahedron with Be-P distances of 2.22 Å. For the purpose of identifying the sequence of phase transformation for Be_3_P_2_, the structures synthesized in experiments must be taken into consideration. Moreover, the calculated lattice parameters of Be_3_P_2_-*Ia*-3 are *a* = *b* = *c* = 10.193 Å at 0 GPa, which is in good agreement with the theoretical (10.19 Å [16,17]) and experimental (10.15 Å [18,19]) values. However, we predicted the structure Be_3_P_2_-*P*-42_1_*m* to have lower energy. The predicted structure of Be_3_P_2_-*P*-42_1_*m* is easier to synthesize by experiment due to its lower formation enthalpy.

As the pressure increased to 33.2 GPa, Be_3_P_2_-*P*-42_1_*m* transforms into a monoclinic Be_3_P_2_-*C*2*/m* structure, with lattice parameters of *a* = 5.624 Å, *b* = 3.283 Å, and *c* = 5.618 Å (Figure 3b). The volume of the Be_3_P_2_-*C*2*/m* structure is 98.64 Å^3^, which demonstrates that the volume decreases by about 23.6% with increasing pressure. Six Be atoms occupy the Wyckoff 4*i* (0.371, 0.000, 0.114) and 2*d* (0.000, 0.500, 0.500) sites, and four P atoms lie in the 4*i* (0.240, 0.000, 0.723) sites in the unit cell. Four Be atoms are coordinated to four P atoms with a Be-P distance of 2.080 Å and two Be atoms are coordinated to six P atoms with a Be-P distance of 2.232 Å. As the pressure increases, three metastable structures, with space groups *Ibam* (Figure 3c), *Cmcm* (Figure 3d), and *C*2/*m* (Figure 3e) at 81.7 GPa, 82.7 GPa, and 90.4 GPa, have been uncovered. For the BeP_2_ phase, at ambient pressure, the optimized structure of BeP_2_ has a tetragonal *I*4_1_/*amd* structure, which is agreement with the reported theoretical data [40] in Figure 3f, with lattice constants of *a* = *b* = 3.582 Å and *c* = 14.994 Å. The volume of the BeP_2_-*I*4_1_/*amd* structure is 192.34 Å^3^, which indicates that the volume increases by about 49.1% compared with the predicted Be_3_P_2_-*P*-42_1_*m* phase. Four Be atoms occupy the Wyckoff 4*a* (0.500, −0.500, 0.500) sites, and eight P atoms lie in the 8*e* (1.000, 0.000, 0.672) sites with four formula units per unit cell. The lattice constant of the c axis is much larger than the a axis and b axis. Each Be atom is coordinated to four P atoms forming a tetrahedron with the Be-P distance of 2.137 Å. Further, these tetrahedrons are interlinked by sharing vertex P atoms that comprise chains with a distance between P and P atoms of 2.283 Å. At higher pressure, BeP_2_-*I*4_1_/*amd* undergoes the phase transition sequence of *I*4_1_/*amd* → *P*4_3_2_1_2 → *Imma*. However, the two novel phases of BeP_2_-*P4_3_2_1_2* and BeP_2_-*Imma* are metastable structures under high pressure. 

**Figure 4:**
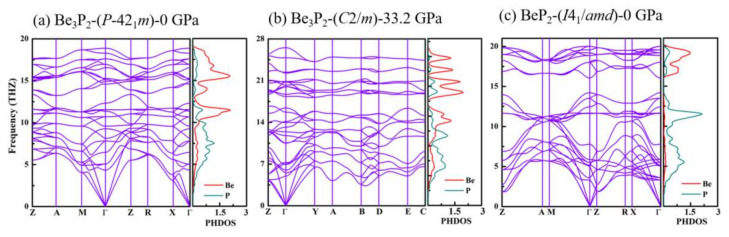
(color online). Phonon−dispersion contours and PHDOS projected on Be and P atoms for (**a**) Be_3_P_2_−*P*−42_1_*m* at 0 GPa, (**b**) Be_3_P_2_−*C*2/*m* at 33.2 GPa, and (**c**) BeP_2_−*I*4_1_/*amd* at 0 GPa.

It is of great importance to research the dynamical stability of these predicted novel structures. The acquired phonon dispersion curves and projected phonon density of states (PHDOS) of the Be_3_P_2_-*P*-42_1_*m*, Be_3_P_2_-*C*2*/m,* and BeP_2_-*I*4_1_/*amd* phases in the pressure range of 0–100 GPa are described in Figure 4. The novel phases are dynamically stable in their accessible pressures since no imaginary vibrational modes are observed in the Brillouin zone. For Be_3_P_2_, at ambient pressure, the maximum optical branch frequency of Be_3_P_2_-*P*-42_1_*m* occurs at 18.8 THz. Under high pressure, the maximum optical branch frequency increases to 26.3 THz in the Be_3_P_2_-*C*2*/m* phase. For BeP_2_, the maximum optical branch frequency of BeP_2_-*I*4_1_*/amd* is 20 THz at 0 GPa. The Be atoms are observed to contribute to all medium and high frequencies (10.2–18.8 THz for Be_3_P_2_-*P-*42_1_*m*, 14.2–26.3 THz for Be_3_P_2_-*C*2*/m*, and 16.2–20 THz for BeP_2_-*I*4_1_*/amd*). Moreover, the P atoms contribute to the low-frequency region (0–10.2 THz for Be_3_P_2_-*P-*42_1_*m*, 0–14.2 THz for Be_3_P_2_-*C*2*/m*, and 0–13.9 THz for BeP_2_-*I*4_1_*/amd*). The relative atomic mass of the Be atom is smaller than that of the P atom, which explains the rationality of the contribution range of Be and P atoms. 

### 3.2. Electronic Properties

**Figure 5:**
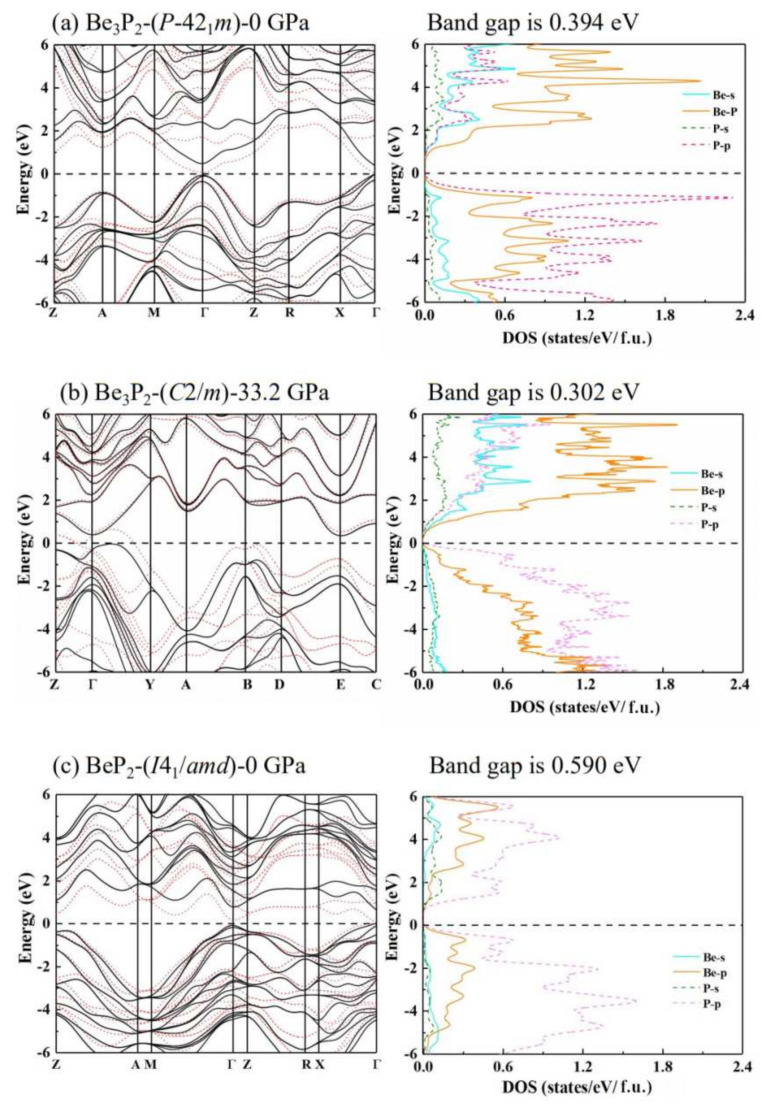
(color online). Electronic band structures and PDOS for (**a**) Be_3_P_2_−*P*−42_1_*m* at 0 GPa, (**b**) Be_3_P_2_−*C*2*/m* at 33.2 GPa, and (**c**) BeP_2_−*I*4_1_*/amd* at 0 GPa. Note that zero energy is on the Fermi level. The dotted (red) and solid (black) lines represent results obtained using the PBE and HSE functional, respectively.

To investigate the potential application of the predicted novel structures of Be_3_P_2_-*P-*42_1_*m*, Be_3_P_2_-*C*2*/m*, and BeP_2_-*I*4_1_*/amd*, we have calculated the electronic band structures and projected density of states (PDOS). As we know, the PBE function underestimates the band gap, so we use the hybrid HSE03 functional to get more accurate band structures. The results are presented in Figure 5; in the band structures, the dotted (red) and solid (black) lines indicate the results obtained using the PBE and HSE functions, respectively. The calculations show that the Be_3_P_2_-*P-*42_1_*m* structure is a semiconductor with a direct bandgap of 0.394 eV at ambient pressure (Figure 5a). With increasing pressure, the bandgap of the Be_3_P_2_-*C*2*/m* phase decreases to 0.302 eV at 33.2 GPa. Unlike in the Be_3_P_2_-*P-*42_1_*m* phase, the Be_3_P_2_-*C*2*/m* phase is an indirect bandgap semiconductor, since the conduction band minimum and valence band maximum are located at the Γ point and between the Γ and Y points in the Brillouin zone, respectively (Figure 5b). For the BeP_2_-*I*4_1_*/amd* phase, it is a semiconductor with a direct bandgap of 0.590 eV at ambient pressure, which is wider than that of the Be_3_P_2_-*P-*42_1_*m* phase. All three phases are excellent semiconductor materials.

For the Be_3_P_2_-*P-*42_1_*m* and Be_3_P_2_-*C*2*/m* phases, the electronic DOS at the Fermi level consists primarily of the p states of Be and P atoms with moderate mixing with the s states of the Be and P atoms. For the results, the orbitals of Be-2s and P-3p as well as Be-2p and P-3s are hybridized formed by covalent bonding. At the Fermi level, the peak value of DOS in the Be_3_P_2_-*P-*42_1_*m* structure is higher than that of the Be_3_P_2_-*C*2*/m* phase since the symmetry of Be_3_P_2_ decreases with increasing pressure. For the BeP_2_-*I*4_1_*/amd* phase, the P-3s states are located at base of the conduction band, as well as the P-3p and Be-2p states, which make important contributions near the Fermi level. The DOS of these compounds once again proves their semiconducting properties.

**Figure 6:**
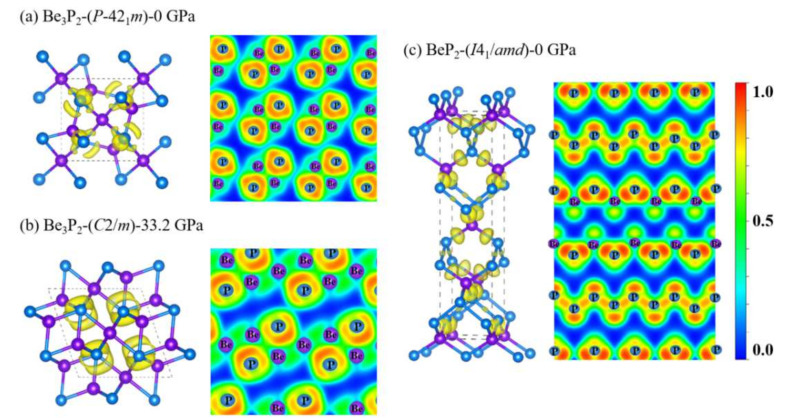
(color online). The ELF graphs for the structures of (**a**) Be_3_P_2_−*P*−42_1_*m*, (**b**) Be_3_P_2_−*C*2/*m*, and (**c**) BeP_2_−*I*4_1_/*amd* with isosurface of 0.8.

The electronic localization functions (ELFs), a measurement of comparative electron localization is computed for the sake of visualization of bonding characteristic of the predicted novel structures of Be_3_P_2_-*P-*42_1_*m*, Be_3_P_2_-*C*2*/m*, and BeP_2_-*I*4_1_*/amd* in Figure 6. Large ELF values (>0.5) indicate a strong tendency for electron pairing, manifesting the production of covalent bonds, whereas tiny ELF values (<0.5) indicate non/less electron localization, revealing the existence of ionic bonds between atoms [41]. For the three novel phases, the ELF maximum, which is forcefully towards the P atoms, shows the polar covalent bonding mutual effect of Be and P atoms. In Figure 6c, the strong electron localization between P and P atoms indicates covalent bonding shown in the polymeric chains. At the same time, the layered structure of BeP_2_-*I*4_1_*/amd* can also be proved from the ELF. 

To describe the electron transfer circumstances more clearly, the calculation of Bader charge transfer is carried out shown in Table 1. It can be identified that charge is transferred from Be to P in the Be_3_P_2_-*P*-42_1_*m*, Be_3_P_2_-*C*2/*m*, and BeP_2_–*I*4_1_/*amd* structures. Our calculations showed that P atoms gained 2.305*e* in Be_3_P_2_-*P*-42_1_*m* and 2.344*e* in Be_3_P_2_-*C*2/*m*. By contrast, P atoms merely acquired a minor value of 0.765*e* for BeP_2_–*I*4_1_/*amd*. Generally, the charge transfer is not an integer and the number of charge transfers is significantly less than the normal integer value. The non-integer charge is calculated from its zero-flux surface due to the space in which the charge is separated. Therefore, the chemical valence of Be and P atoms is +2 and −3 in the predicted two phases of Be_3_P_2_-*P-*42_1_*m* and Be_3_P_2_-*C*2*/m*, respectively. For the BeP_2_-*I*4_1_*/amd* phase, Bader analysis reveals the chemical valence of Be and P atoms is +2 and −1. The various chemical valences of P are determined by the valence electron configuration of 3s^2^3p^3^. The calculation results of Bader charge transfer support the above analysis of electron local function and structure. 

## 4. Conclusions

In conclusion, we used first-principles developmental crystal structure forecasting to investigate the crystal structures and probable stoichiometries in the Be-P system. The BeP_2_-*I*4_1_*/amd* structure has been resoundingly duplicated at ambient pressure, confirming the dependability of our calculations regarding binary Be-P compounds. We find that the Be_3_P_2_-*P-*42_1_*m* and BeP_2_-*I*4_1_*/amd* structures exist at ambient pressure, as well as a new phase that is Be_3_P_2_-*C*2*/m* at 33.2 GPa. The predicted structures, except for the aforementioned three structures, are unsteady within the range of pressures we investigated. The theoretical phonon dispersion curves prove the dynamical stability of existing expected structures. Electronic structure calculations show that predicted novel structures of Be_3_P_2_-*P-*42_1_*m*, Be_3_P_2_-*C*2*/m*, and BeP_2_-I4_1_/amd are all excellent semiconductor materials, which have bandgaps of 0.394 eV, 0.302 eV, and 0.590 eV, respectively. Moreover, the charge accumulation along the bonding directions between Be and P is an indication of polar covalent bonding. The Bader charge analysis illustrates those electrons obviously transferred from Be to P atoms in these newfangled phases and indicates that the chemical valence of P atom varies with the stoichiometries. Our findings should stimulate further theoretical and experimental research into the high-pressure development of crystal structures and electrical characteristics in Be-P systems.

## Figures and Tables

**Table 1:** The Bader charge transfer of Be_3_P_2_−*P*−42_1_*m*, Be_3_P_2_−*C*2/*m*, and BeP_2_−*I*4_1_/*amd*.

Phase	Pressure (GPa)	Atom	Number	Charge Value (*e*)	δ(*e*)
Be_3_P_2_-*P*-42_1_*m*	0 GPa	Be1	4	0.466	−1.534
-	-	Be2	2	0.457	−1.543
-	-	P1	4	7.305	2.305
Be_3_P_2_-*C*2/*m*	33.2 GPa	Be1	4	0.452	−1.548
-	-	Be2	2	0.408	−1.592
-	-	P1	4	7.344	2.344
BeP_2_-*I*4_1_/*amd*	0 GPa	Be1	4	0.470	−1.530
-	-	P1	8	5.765	0.765

## Data Availability

Data sharing is not applicable to this article.
